# Use of Tobacco and Alternative Nicotine Products Among People with HIV: A Cross-Sectional Multicenter Survey

**DOI:** 10.1007/s10461-025-04958-7

**Published:** 2025-11-13

**Authors:** Andrea De Vito, Andrea Giacomelli, Maria Mazzitelli, Gianmaria Baldin, Massimiliano Fabbiani, Agnese Colpani, Miriam Galimberti, Andrea Carbone, Aurora Civati, Annamaria Cattelan, Simona Di Giambenedetto, Giordano Madeddu

**Affiliations:** 1 Unit of Infectious Diseases, Department of Medicine, Surgery and Pharmacy, University of Sassari, Sassari, Italy; 2 Department of Infectious Diseases Unit, ASST Fatebenefratelli Sacco, Milan, Italy; 3 Department of Biomedical and Clinical Sciences, Università Degli Studi di Milano, Milan, Italy; 4 Infectious and Tropical Diseases Unit, Padua University Hospital, 35128 Padua, Italy; 5 Unit of Infectious Diseases, Fondazione Policlinico Universitario A. Gemelli IRCCS, Rome, Italy; 6 Catholic University of the Sacred Heart, Rome, Italy; 7 Department of Medical Biotechnologies, University of Siena, Siena, Italy; 8 Infectious and Tropical Diseases Unit, Azienda Ospedaliero-Universitaria Senese, Siena, Italy

**Keywords:** HIV, Smoking, Tobacco harm reduction, Electronic cigarettes, Heated tobacco products, Lung cancer screening

## Abstract

People with HIV (PWH) have a higher prevalence of smoking compared to the general population, which contributes to an increased risk of non-AIDS-related comorbidities. Heated tobacco products (HTPs) and electronic cigarettes (e-cigarettes) have added complexity to tobacco use behaviors. This study aimed to investigate smoking habits, including HTPs and e-cigarettes use and to describe patterns of tobacco use. We conducted a cross-sectional survey among PWH attending five Italian University Hospitals. An anonymous questionnaire collected information on smoking status, history, use of HTPs and e-cigarettes, motivations for product use, and engagement with smoking cessation services and lung health screenings. A total of 1,081 PWH were included: 41.9% were current smokers (CS), 31.5% former smokers (FS), and 26.6% never smokers. CS reported a median consumption of 15 cigarettes per day and a smoking duration of 30 years. HTP use was reported by 10.6% of participants, most commonly among FS and CS, while e-cigarette use was reported by 15.9%, with the highest prevalence among younger individuals. Dual use of cigarettes and e-cigarettes was found in 21.0% of CS. The main reasons for e-cigarette use were smoking reduction (75%) and perceived lower harm (53.5%). Only 5% of participants accessed smoking cessation services, and 60.5% of CS had attempted to quit, but physician recommendations for cessation support were infrequent (21.0%). Lung health screening was underutilized, with only 17.0% undergoing a CT scan and 12.7% a pulmonology consultation. These findings underscore the need for targeted strategies to address tobacco use and improve preventive care in PWH.

**Supplementary Information:**

The online version contains supplementary material available at 10.1007/s10461-025-04958-7.

## Introduction

Smoking remains one of the leading modifiable causes of morbidity and mortality worldwide, contributing significantly to the global burden of disease [1]. People with HIV (PWH) are disproportionately affected by tobacco use, with smoking prevalence rates among PWH being nearly double compared to the general population [2]. This increased prevalence amplifies the already elevated health risks faced by PWH, who experience higher rates of non-AIDS-related comorbidities and complications, including cardiovascular diseases, respiratory illnesses, and malignancies [3, 4].

One of the most concerning health risks associated with smoking in PWH is the heightened incidence of lung cancer. Several studies have demonstrated that PWH are at a significantly greater risk of developing lung cancer compared to people without HIV, even after adjusting for smoking intensity and duration [5–8]. The immunosuppressive effects of HIV, chronic inflammation, and the potential carcinogenic interactions between HIV and tobacco compounds may contribute to further increase this risk [9]. Moreover, smoking-related cancers in PWH often present at a younger age and with more aggressive clinical features, further worsening treatment outcomes [8, 10]. In Italy, smoking has been consistently associated with a higher burden of non-AIDS comorbidities among PWH. De Socio et al. reported that more than half of Italian PWH were active smokers, a prevalence nearly double that of the general population, and that smoking cessation rates were markedly lower in PWH compared to HIV-negative individuals [11]. Other Italian cohort studies have highlighted the contribution of tobacco use to cardiovascular diseases, chronic obstructive pulmonary disease, and malignancies in this population, further compounding the risk already associated with HIV-related inflammation and immune dysregulation. These findings underscore the need to characterize better smoking behaviors and emerging nicotine use patterns in Italian PWH, to inform prevention and tailored cessation strategies.

The advent of harm reduction strategies, particularly heated tobacco products (HTPs) and electronic cigarettes (e-cigarettes), introduced a new dynamic into tobacco use behaviors. Despite initially being marketed as safer alternatives to conventional cigarettes, these devices have been widely adopted by smokers seeking to reduce or quit tobacco use [12–15]. However, growing evidence challenges the perception of e-cigarettes and HTPs as alternatives without health consequences [16–18]. Recent studies highlight that while e-cigarettes may deliver fewer carcinogens compared to traditional cigarettes, they are not without risks. For instance, vaping has been associated with adverse cardiovascular and respiratory outcomes, and concerns persist regarding the potential long-term carcinogenicity of aerosolized nicotine and other toxicants [19–21].

Moreover, the combined use of conventional cigarettes and e-cigarettes exacerbates health risks. A case-control study by Bittoni et al. revealed that people who both smoked and vaped had a fourfold higher risk of developing lung cancer compared to those who were exclusive smokers, underscoring the cumulative harm of the combined use [22]. This is particularly concerning for PWH, given their heightened baseline susceptibility to lung cancer due to chronic inflammation and HIV-related immune dysregulation.

In PWH, the use of e-cigarettes and HTPs introduces additional complexity. A study by Thorne et al. found that 5.9% of PWH currently use e-cigarettes, with a higher prevalence among current smokers and people with mental health comorbidities [23]. This suggests that e-cigarettes are not only used as cessation aids but may also perpetuate nicotine dependence in this population. Compounding these issues, Wang et al.. demonstrated that e-cigarette users eligible for lung cancer screening were less likely to undergo screening, potentially delaying the diagnosis of lung cancer in high-risk groups [24].

Despite the exponentially increasing adoption of e-cigarettes and HTPs, there is limited data on their impact on smoking behaviors and associated cancer risks among PWH. Given the high prevalence of smoking in this population and the emerging concerns surrounding alternative tobacco products, it is critical to understand the patterns and implications of e-cigarette and HTP use in PWH. Therefore, this study aims to investigate smoking habits, including the use of HTPs and e-cigarettes, among people with HIV in Italy, with particular attention to usage patterns, drivers of alternative product use, and access to cessation and lung health services.

## Methods

We conducted a cross-sectional survey-based study on smoking habits among PWH aged ≥ 18 years across five Italian University Hospitals (University Hospital of Sassari, IRCCS A. Gemelli - Rome, ASST_Fatebenefratelli Sacco - Milan, University Hospital of Siena, and University Hospital of Padua). An anonymous online survey was created using Google Forms, and the link was converted into a QR code, which was displayed in the participating outpatient clinics from November 1, 2024, to February 5, 2025. PWH attending the outpatient clinics who were able to use electronic devices could freely scan the QR code and complete the survey. For individuals who were unable to use their devices, a printed paper version of the survey was administered.

The first page of the questionnaire included the study rationale, privacy regulations, and a consent form. If people declined participation, the survey ended immediately without further questions. For those who agreed to participate, no personal identifying data was collected. To ensure participant anonymity, age and years living with HIV were categorized into six-year ranges for age and four-year ranges for years living with HIV. Gender was categorized as cisgender male/female, transgender male/female, or non-binary. An “I prefer not to answer” option was provided for all three questions.

Regarding smoking habits, we assessed whether participants were current or former smokers, the number of cigarettes they smoked/smoke daily, and the duration of smoking. The research team calculated pack-years after the survey was completed. To contextualize smoking exposure, we used two established thresholds for cumulative tobacco consumption: 20 pack-years and 30 pack-years. The 20-pack-year cut-off was chosen based on the screening criteria recommended by the U.S. Preventive Services Task Force (USPSTF) and other American guidelines, whereas the 30-pack-year threshold aligns with the screening recommendations in Europe [25, 26]. These cut-offs were used to evaluate the distribution of heavy smoking exposure across different age groups.

We also investigated the use of HTPs and e-cigarettes. For HTPs, participants were asked how many consumables they used daily and how long they had used the product. For e-cigarettes, we inquired about the nicotine concentration in the e-liquid and the daily amount of liquid consumed. Additionally, we explored whether participants used both traditional cigarettes and e-cigarettes simultaneously and the reasons for initiating the use of these devices.

Finally, we assessed whether participants had ever tried to quit smoking, accessed anti-smoking clinics, or received advice from their doctor to attend such services. We also asked if healthcare providers had prescribed lung evaluations, including CT scans, X-rays, spirometry, or pulmonology consultations.

Uncompleted answers have been excluded from the analysis (i.e., number of cigarettes, years of smoking).

### Statistical Analysis

A convenience sample was used, including all PWH who consecutively and voluntarily chose to participate. Qualitative variables were presented as absolute frequencies and percentages, while quantitative variables were reported as means with standard deviations or medians with interquartile ranges (IQR), depending on data distribution. The Shapiro-Wilk test was used to assess normality.

Differences in qualitative variables were analyzed using the Chi-square test or Fisher’s exact test when appropriate. For quantitative variables, we used the one-way ANOVA for normally distributed data and the Kruskal-Wallis test for non-normally distributed data. Post-hoc analyses included Bonferroni correction for normally distributed variables and Dunn’s post-hoc pairwise comparisons with Šidák correction for non-normally distributed data. A p-value < 0.05 was considered statistically significant. All analyses were performed using STATA version 16.1 (StatsCorp, TX, USA).

### Ethical Approval

The study was conducted in accordance with the Declaration of Helsinki and approved by each local Institutional Ethics Committee (protocol number of the promoting centre: 5284/15).

## Results

One thousand two hundred and thirty-three PWH gave their consent and participated in the survey; 152 were excluded from the final analysis due to missing information. Among 1,081 PWH included, 288 (26.6%) identified as never tobacco smokers (NS), 453 (41.9%) as current smokers (CS), and 340 (31.5%) as former smokers (FS). Data are summarized in Table 1).

**Table 1:** Demographic characteristics and traditional smoking habits among 1,081 people living with HIV, stratified by smoking status (never tobacco smokers, former smokers, and current smokers)

Variables name	Never smoker (*n* = 288)	Current smoker (*n* = 453)	Former smoker (*n* = 340)	Total (*n* = 1081)	Χ²	*p*-value
Age						
< 20 years, n (%)	6 (2.1)	2 (0.4)	6 (1.8)	14 (1.3)	19.71	0.032
20–30 years, n (%)	17 (5.9)	19 (4.2)	14 (4.1)	50 (4.6)		
31–40 years, n (%)	35 (12.1)	64 (14.1)	39 (11.5)	138 (12.8)		
41–50 years, n (%)	65 (22.6)	99 (21.9)	63 (18.5)	227 (21.0)		
51–60 years, n (%)	100 (34.7)	150 (33.1)	99 (29.1)	349 (32.3)		
> 60 years, n (%)	65 (22.6)	119 (26.3)	119 (35.0)	303 (28.0)		
Gender						
Cisgender women, n (%)	63 (21.9)	133 (29.4)	104 (30.6)	300 (27.7)	15.53	0.050
Transgender women, n (%)	10 (3.5)	9 (2.0)	11 (3.2)	30 (2.8)		
Cisgender men, n (%)	211 (73.3)	299 (66.0)	212 (62.4)	722 (66.8)		
Transgender men, n (%)	1 (0.3)	1 (0.2)	0	2 (0.2)		
Non-Binary, n (%)	3 (1.0)	11 (2.4)	13 (3.8)	27 (2.5)		
Years living with HIV						
< 5 years, n (%)	58 (20.1)	63 (13.9)	44 (12.9)	165 (15.3)	21.13	0.002
5–10 years, n (%)	54 (18.8)	70 (15.5)	48 (14.1)	172 (15.9)		
11–20 years, n (%)	87 (30.2)	120 (26.5)	87 (25.6)	294 (27.2)		
> 20 years, n (%)	89 (30.9)	200 (44.1)	161 (47.4)	450 (41.6)		
Smoking habits						
Cigarettes per day, median (IQR)	–	15 (10–20)	15 (10–20)	15 (10–20)	15.39	< 0.001
Years of smoking, median (IQR)	–	30 (20–40)	20 (10–30)	25 (14–35)	81.20	< 0.001
Pack-years, median (IQR)	–	18 (7.5–30)	12.5 (5–29)	15 (6–30)	8.21	0.004
Pack-years ≥ 20, n(%)	–	220 (48.6)	130 (38.2)	350 (32.4)	1.34	< 0.001

More than 60% of participants were over 50 years. FS were generally older, with 35.0% of people being over 60 years of age, compared to 26.3% of CS and 22.6% of NS.

In terms of gender, the majority were cisgender men (66.8%), followed by cisgender women (27.7%). A small percentage identified as transgender women (2.8%) or non-binary (2.5%). NS had a higher proportion of cisgender men (73.3%) compared to CS (66.0%) and FS (62.4%).

The duration of known HIV infection varied significantly between groups (*χ²* = 21.13; *p* = 0.002). FS had the longest history, with 47.4% living with HIV for over 20 years, compared to 44.1% and 30.9% of CS and NS, respectively.

Among CS, the median number of cigarettes smoked per day was 15 (IQR: 10–20), likewise, FS before quitting. However, CS had a longer smoking history (30 years, IQR: 20–40) compared to FS (20 years, IQR: 10–30) (*χ²* = 80.47, *p* < 0.001). Additionally, number of pack-years median was significantly higher among CS (18, IQR: 7.5–30) than FS (12.5, IQR: 5–29) (*χ²* = 8.19, *p* = 0.004) (Table 1).

No people belonging to age groups < 20 and 20–30 accumulated at least 20 pack-years. Among participants aged 31–40 years, only 7.8% reached this threshold, and the proportion increased progressively with age. In particular, 28.4% of people aged 41–50 years, 52.2% of those aged 51–60 years, and 68.1% of those over 60 years had a cumulative smoking exposure of at least 20 pack-years. When considering a higher threshold of at least 30 pack-years, the same age-related trend was observed, with only 1.0% of those aged 31–40 years meeting this criterion, increasing to 12.3% in the 41–50 age group, 31.7% in those aged 51–60 years, and reaching 48.3% in individuals over 60 years. Overall, 27.1% of participants accumulated at least 30 pack-years (Table S1). No differences have been seen between the genders (Table S2).

Considering the use of HTPs, 115 (10.6%) participants reported using them, with significant differences across smoking groups (*χ²* = 30.50, *p* < 0.001). HTP use was most common among FS (49, 14.4%) and CS (60, 13.3%), while it was quite rare among NS (6, 2.1%) (Table 2). Regarding the number of sticks used per day, there was no difference among NS, CS, and FS (*χ²* = 5.18, *p* = 0.075). Dunn’s post-hoc pairwise comparisons with Šidák correction revealed a marginally significant difference between FS and CS (*Z-value* = −2.18, *p* = 0.043) (Table S3).

**Table 2:** Characteristics of new smoking habits among 1,081 people living with HIV, stratified by tobacco smoking status (never tobacco smokers, former smokers, and current smokers)

Variables name	Never smoker (*n* = 288)	Current smoker (*n* = 453)	Former smoker (*n* = 340)	Total (*n* = 1081)	Χ²	*p*-value
Heated tobacco, n (%)	6 (2.1)	60 (13.2)	49 (14.4)	115 (10.6)	30.50	< 0.001
Number of consumables per day, median (IQR)	7.5 (5–15)	10 (5–15)	10 (10–20)	10 (5–15)	5.18	0.075
Electronic cigarette, n (%)	15 (5.2)	98 (21.6)	59 (17.3)	172 (15.9)	36.27	< 0.001
Quantity of nicotine (mg/mL), n (%)*					19.81	0.031
Don’t know	5 (33.3)	41 (41.8)	16 (27.1)	62 (36.0)		
Without nicotine	8 (53.3)	12 (12.2)	12 (20.3)	32 (18.6)		
< 5 mg	1 (6.6)	19 (19.4)	12 (20.3)	32 (18.6)		
5–10 mg	1 (6.6)	19 (19.4)	16 (27.1)	36 (20.9)		
> 10 mg	0 (0.0)	7 (7.2)	3 (5.2)	10 (5.81)		
Quantity of liquid smoked daily (mL), n (%)*					9.74	0.283
Don’t know	10 (66.7)	64 (65.3)	35 (59.2)	109 (63.4)		
< 2mL	4 (26.7)	14 (14.3)	5 (8.5)	23 (13.4)		
2-4mL	0	12 (12.2)	8 (13.6)	20 (11.6)		
4.1-8mL	1 (6.6)	5 (5.1)	6 (10.2)	12 (7.0)		
> 8mL	0	3 (3.1)	5 (8.5)	8 (4.6)		
Smoking together Tobacco and electronic cigarettes, n (%)	–	95 (21.0)	17 (5.0)	112 (14.1)	84.92	< 0.001
Reasons for initiating electronic cigarette use, n (%)						
To reduce smoking	0	72 (73.5)	57 (97.7)	129 (75)	24.89	< 0.001
Curiosity	9 (60.0)	23 (23.5)	10 (17.2)	42 (24.4)	14.55	< 0.001
Perceived as less harmful	5 (33.3)	34 (34.7)	53 (91.4)	92 (53.5)	25.69	< 0.001
To smoke indoors	0	23 (23.5)	21 (35.6)	44 (25.6)	5.78	0.056
Better scent	0	16 (16.3)	19 (32.2)	35 (20.3)	4.84	0.089
Not specified	4 (26.7)	17 (17.3)	5 (8.5)	26 (15.1)	5.32	0.070
Less expensive	0	10 (10.2)	9 (15.2)	19 (11.0)	2.17	0.338
Other	1 (6.6)	2 2.0)	2 (3.4)	5 (2.9)	1.18	0.554

*IQR* Interquartil Range, *Data on 172 participants

Regarding e-cigarette use, 172 (15.9%) participants reported current use, of whom 98 (21.6%) were CS, 59 (17.3%) were FS, and 15 (5.2%) were NS (χ² = 36.27, *p* < 0.001). Analysis of nicotine concentrations revealed that FS used moderate nicotine levels (5–10 mg/mL, 16/59, 27.1%) most frequently, and a notable proportion opted for nicotine-free e-cigarettes (12/59, 20.3%). CS predominantly used nicotine-containing products, with 19/98 (19.4%) using moderate (5–10 mg/mL) and the same proportion using low (< 5 mg/mL) concentrations. Among people who never smoked, the majority (8/15, 53.3%) preferred nicotine-free e-cigarettes, highlighting distinct usage patterns across groups (*χ²* = 19.81, *p* = 0.031). Daily e-liquid consumption did not significantly differ between groups (*χ²* = 9.74, *p* = 0.283), with 109 (63.4%) of participants across all categories selecting “Don’t know”. Among participants who were able to provide a specific amount, both CS and FS reported similar consumption, mainly between < 2 mL and 2–4 mL per day.

The combined use of heated tobacco and e-cigarettes was reported by 6.1% of participants, particularly among CS (8.4%) and FS (7.0%).

HTP use was most prevalent in people under 20 years (50.0%) and it gradually decreased by age, reaching the lowest prevalence in over 60 years (4.3%). Similarly, e-cigarette use shows the highest prevalence among individuals under 20 years (35.7%), with a notable decline in older age groups, indicating a greater inclination towards e-cigarettes among the youngest PWH (Table S4-S5).

Regarding the HTP use in different genders, it was highest among non-binary individuals (33.3%) and transgender women (30.0%), while cisgender men showed the lowest prevalence (8.7%). Similarly, e-cigarette use was most common among non-binary individuals (33.3%) and transgender men (50.0%) (Table S6-S7).

Drivers for e-cigarette use varied by smoking status. Reducing conventional smoking was the most reported reason, acknowledged by 73.5% of CS and 97.7% of FS. Perceiving e-cigarettes as less harmful was also common (34.7% and 91.4%, respectively). Curiosity influenced e-cigarette use in 60% of people who never smoked and in 23.5% of CS.

Other reasons included the possibility of smoking indoors (23.5% of CS and 35.6% of FS), better scent (16.3% and 32.2%), and lower cost (10.2% and 15.22%). NS were less likely to cite harm reduction or smoking cessation as motivation, with curiosity being their primary driver.

A significant proportion (60.5%) of CS attempted to quit, but only a small fraction accessed smoking cessation services (4.4% of CS and 5.9% of FS). The amount of medical referrals to a smoking cessation center was significantly higher (*χ²* = 22.79, *p* < 0.001) in CS (21.0%) than in FS (8.5%).

Regarding smoking-related medical assessments and recommendations, CS reported a significantly higher percentage than FS of prescribed indications to perform instrumental investigations (30.7% vs. 21.2%, χ² = 8.99, *p* = 0.003) or chest CT scan (19.4% vs. 13.8%, *χ²* = 4.32, *p* = 0.038). Similarly, a higher rate of pulmonary consultations (16.1% vs. 8.2%, *χ²* = 10.85, *p* = 0.001) and spirometry tests (11.0% vs. 5.6%, *χ²* 7.26, *p* = 0.007) was reported by CS compared to FS (Table 3).

**Table 3:** Smoking cessation efforts and smoking-related medical evaluations among current and former smokers living with HIV

Variables name	Current smoker (*n* = 453)	Former smoker (*n* = 340)	Total (*n* = 793)	Χ²	*p*-value
Tried to Quit Smoking, n (%)	274 (60.5)	340 (100)	614 (77.4)	173.51	< 0.001
Accessed Smoking Cessation Service, n (%)	20 (4.4)	20 (5.9)	40 (5.0)	0.87	0.350
Doctor Recommended Cessation Center, n (%)	95 (21.0)	29 (8.5)	124 (15.6)	22.79	< 0.001
Doctor Prescribed Tests for Smoking, n (%)	139 (30.7)	72 (21.2)	211 (26.6)	8.99	0.003
Chest CT Scan, n (%)	88 (19.4)	47 (13.8)	135 (17.0)	4.32	0.038
Chest X-ray, n (%)	50 (11.0)	29 (8.5)	79 (10.0)	1.36	0.243
Pulmonary Consultation, n (%)	73 (16.1)	28 (8.2)	101 (12.7)	10.85	0.001
Spirometry, n (%)	50 (11.0)	19 (5.6)	69 (8.7)	7.26	0.007

**Fig. 1:**
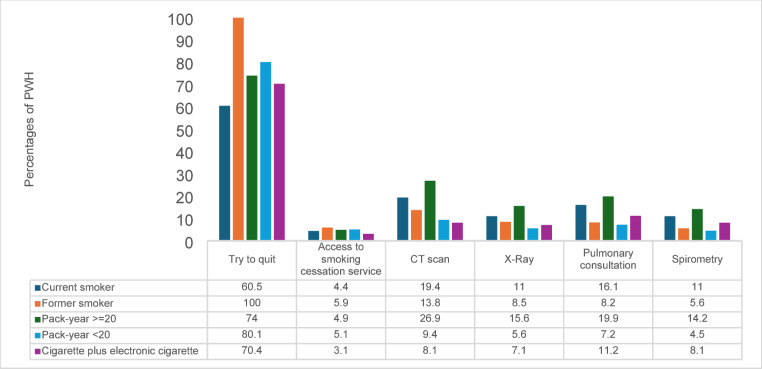
Smoking cessation attempts and utilization of smoking-related health services among PWH across smoking subgroups

Participants who consumed ≥ 20 pack years were more likely to have undergone CT scans (26.9%) and pulmonary consultations (19.9%). Notably, people who had a combined use of cigarettes and e-cigarettes showed a moderate rate of quitting attempts (70.4%) but had the lowest access to cessation services (3.1%) (Fig. 1).

## Discussion

This study provides a comprehensive analysis of smoking patterns in PWH in Italy, highlighting both traditional and emerging tobacco product use. Our findings confirm that a significant proportion of PWH are current smokers (41.9%), while 31.5% are former smokers, and 26.6% have never smoked. Additionally, we observed a notable prevalence of HTP and e-cigarette use. The prevalence of CS in our cohort of PWH is more than double the estimated 19.5% smoking rate in the general Italian population, according to the ISTAT 2023 data [27]. Our findings align with those of De Socio et al., published in 2020, who reported an even higher smoking prevalence of 51.6% among PWH, further confirming the elevated tobacco use in this population compared to the 25.9% prevalence in the general Italian population [11]. Similarly, the prevalence of former smokers in our cohort was 31.5%, which is lower than the 50.1% reported in the general Italian population by ISTAT. In this regard, De Socio et al.. found an even lower quitting rate among PWH, with only 27.1% identifying as former smokers compared to 50.1% in the general population. These findings suggest that smoking cessation is less common among PWH, highlighting potential barriers to quitting, such as higher nicotine dependence or limited access to cessation programs. While national data indicate a steady increase in smoking cessation rates, our results and those of De Socio et al.. confirm that quitting remains a significant challenge for PWH, emphasizing the need for targeted interventions within HIV care settings.

A systematic review reported an overall prevalence of active smoking among PWH on antiretroviral therapy (ART) of 36.1%, with notable regional differences: 45.2% in high-income countries, 30.8% in middle-income countries, and 10.1% in low-income countries​ [28]. In comparison, our study found a similar prevalence of CS, with 41.9% of the 1,081 surveyed PWH identifying as current smokers. Additionally, 31.5% were former smokers, and only 26.6% had never smoked, highlighting a substantial lifetime exposure to tobacco in our cohort.

Regarding the prevalence of e-cigarette use in PWH, despite a higher prevalence found when compared with the study conducted in the U.S. by Thorne et al. (15.9% vs. 5.9%), a similar age-related trend was reported in the two studies [23]; e-cigarette use is most common among younger PWH. We reported that 35.7% of individuals under 20 years and 26% of those aged 20–30 years reported use. Usage declined with age, dropping to 9.6% among those over 60 years. Similarly, the U.S. study showed higher usage rates in the 25–34 age group. Gender-based analysis in our study showed notable differences in e-cigarette use. Transgender men reported the highest use (50%), followed by non-binary individuals (33.3%) and transgender women (23.3%). Cisgender men and women showed lower rates, of 15.7% and 14%, respectively. While the U.S. study identified higher usage among males and non-Hispanic White PWH, it did not explore differences among gender-diverse groups, which emerged as significant in our analysis.

Tobacco harm reduction strategies, such as the use of vaporized nicotine products, have been explored as alternatives for PWH who smoke [29]. While these products may help some individuals reduce or quit smoking, uncertainty remains regarding their long-term safety and efficacy​ [30]. Healthcare providers play a crucial role in supporting smoking cessation among PWH, but barriers such as a lack of confidence in prescribing nicotine replacement therapies and competing clinical priorities often hinder effective intervention​ [31, 32].

A particularly concerning finding in our study was the initiation of HTPs and e-cigarettes among individuals who had never previously smoked conventional tobacco. This highlights the evolving landscape of nicotine use, where alternative products may serve as an entry point into nicotine addiction rather than as cessation aids. Such a trend is troubling, especially considering that even nicotine-free e-cigarettes have been linked to adverse health outcomes, including increased cardiac sympathetic activity and oxidative stress, which elevate cardiovascular risk​.

The data also reveal that a significant portion of PWH who successfully quit smoking conventional cigarettes adopted alternative nicotine delivery systems, such as e-cigarettes and HTPs. While harm reduction strategies often promote these alternatives as safer options, studies have questioned their efficacy and safety. For example, dual users of cigarettes and e-cigarettes have been shown to have a fourfold higher risk of developing lung cancer compared to exclusive smokers of conventional cigarettes [22]​. This raises concerns that transitioning from traditional smoking to alternative products may not eliminate health risks but could potentially compound them. Additionally, emerging evidence underscores cardiovascular risks associated with both heated tobacco products (HTPs) and e‑cigarettes. A randomized crossover study demonstrated that even a single use of HTPs, e‑cigarettes (with or without nicotine), and combustible cigarettes led to significant inflammatory responses, endothelial dysfunction, and increased arterial stiffness [33]. Moreover, a meta‑analysis found that acute e‑cigarette use significantly elevated PWV and AIx75, indicating immediate negative vascular effects [34]. Additional studies suggest that chronic e‑cigarette use impairs endothelial function comparably to conventional tobacco, through mechanisms involving oxidative stress, inflammation, and sympathetic activation [35]. Taken together, these data highlight that alternative nicotine delivery systems may increase the cardiovascular risks. Although our study was not designed to evaluate vascular outcomes directly, these cardiovascular considerations further emphasize the urgency of addressing HTP and e‑cigarette use among PWH, who already bear elevated baseline cardiovascular vulnerability due to HIV-associated inflammation and immune dysregulation [36, 37].

Another critical aspect of the findings is the low engagement in lung health screening and preventive care among PWH, even among those with significant smoking histories. Despite guidelines recommending lung cancer screening for high-risk individuals, uptake remains suboptimal. Wang et al. demonstrated that current e-cigarette users had a 21% lower likelihood of undergoing LCS compared to non-users [38]​. This reluctance or lack of engagement in screening is particularly concerning given the elevated lung cancer risk associated with both traditional and alternative nicotine products.

Moreover, only a minority of participants in this study were referred for pulmonary consultations or lung cancer screenings, such as CT scans or spirometry, despite having substantial smoking histories. This gap in preventive care underscores the need for enhanced clinician awareness and proactive engagement in smoking cessation and screening efforts. It also highlights an opportunity for tailored interventions targeting PWH, a population already at heightened risk for respiratory comorbidities due to HIV and associated immunosuppression.

This study has several limitations that should be acknowledged. First, the cross-sectional design limits our ability to establish causal relationships between smoking behaviors, the use of alternative nicotine products. The reliance on self-reported data introduces the possibility of recall bias and social desirability bias, which may have led to underreporting of tobacco and alternative product use, particularly among current smokers. Conversely, selection bias could also have played a role, as individuals who smoke may have been more motivated to participate in the survey, potentially leading to an overestimation of smoking prevalence. Additionally, the anonymous nature of the survey prevented us from validating self-reported smoking behaviors with clinical data.

Another important limitation is that we did not collect information about participants’ comorbidities and viro-immunological status to maintain strict anonymity. While this approach ensured participant privacy, it also limited our ability to analyze how existing health conditions may influence smoking behaviors or the use of alternative nicotine products. The absence of clinical data, including comorbidities, restricts a deeper understanding of the potential health impacts of tobacco and alternative product use in this population.

Despite these limitations, our study has several strengths. It represents one of the largest investigations into smoking behaviors among PWH in Italy, providing valuable insights into both traditional smoking and the use of emerging nicotine products. Given the limited national data on tobacco and alternative nicotine use in PWH, our study helps fill a critical knowledge gap, offering a more comprehensive understanding of smoking patterns among Italian PWH.

## Conclusion

This study highlights the high prevalence of traditional cigarette use in combination with the adoption of alternative nicotine products, such as e-cigarettes and HTPs. A particularly concerning finding is the initiation of HTPs and e-cigarettes among never-smokers, suggesting that these products may act as a gateway to nicotine addiction rather than serving solely as harm reduction tools. Our findings also underscore the challenges in smoking cessation among PWH, with many former smokers transitioning to alternative nicotine products, thereby maintaining nicotine dependence. The low rates of lung health screening and preventive care further compound the risks faced by this population, despite their elevated susceptibility to smoking-related comorbidities. Public health initiatives should focus on preventing the initiation of HTPs and e-cigarettes, particularly among never-smokers, while also promoting adequate cessation support and increasing access to lung health screenings. These efforts are vital to mitigating the compounded risks of tobacco use and improving long-term health outcomes for PWH.

## Supplementary Information

Below is the link to the electronic supplementary material.

Supplementary Material 1

Supplementary Material 2

## Data Availability

The data collected for this study will be made available to others, and they will be available upon publication of this article and can be accessed in the Supplementary Materials accompanying the manuscript. The data are freely available to anyone without any restrictions.
