# Supplementary material for: Use of Tobacco and Alternative Nicotine Products Among People with HIV: A Cross-Sectional Multicenter Survey
Source: AIDS Behav. 2025 Nov 13;30(4):1088–96. doi: 10.1007/s10461-025-04958-7 (PMC13076392; doi:10.1007/s10461-025-04958-7)
Supplement: Supplementary file 1 — Supplementary Material 1 [file 10461_2025_4958_MOESM1_ESM.docx]

**PART 1**

**Smoking Questionnaire for People Living with HIV**

**Dear Participant,**

We inform you that we are conducting a survey on smoking habits among people living with HIV.

For this reason, we invite you to participate in the survey.

The main objective of the survey is to describe changes in smoking habits among people living with HIV, particularly evaluating the use of alternatives to traditional cigarettes such as electronic cigarettes and devices like IQOS.

The procedure involves completing an online questionnaire, which will take approximately 5 minutes.

Participation is entirely free and voluntary, and there are no risks involved for participants. Choosing not to participate, or withdrawing after initially agreeing, will have no negative consequences for you.

**Processing of Personal Data**

No personal data will be collected, and the survey is completely anonymous.

All personal data acquired during this study will be processed in full compliance with the provisions of Regulation (EU) 2016/679 on **General Data Protection Regulation (GDPR)**, effective from May 25, 2018, and Italian Legislative Decree no. 196 of June 30, 2003 — **Personal Data Protection Code**, as long as it is not repealed by the aforementioned European regulation.

According to this legislation, since the data is collected anonymously, there is no need to appoint a data protection officer.

**Nature of Data and Processing Methods**

All information collected during this survey is confidential and will be handled in compliance with the aforementioned laws.

The data you provide will be anonymous, meaning that the collected material will be anonymized at the source and cannot be traced back to the identity of the survey participant. This material will be analyzed and processed for scientific research purposes only by the personnel responsible for conducting the survey.

The data, processed using electronic tools as well, may be shared in strictly anonymous form through meetings, conferences, and scientific publications. In any case, we reiterate that the data will be anonymous and in no way traceable to the participant’s identity. The data may only be presented in aggregated form.

We thank you for your time and cooperation.

**Please select only one option:**
☐ I consent to participate

**PART 2**

**1. How old are you? ***

*Select only one answer*

- Under 20 years
- 21-30 years
- 31-40 years
- 41-50 years
- 51-60 years
- Over 60 years
- I prefer not to answer

**2. What is your gender identity? ***

*Select only one answer*

- Man
- Woman
- Transgender woman
- Transgender man
- I do not identify with a binary gender
- Other
- I prefer not to answer

**3. How many years have you been living with HIV?**

*Select only one answer*

- Less than 5 years
- 5-10 years
- 10-20 years
- More than 20 years

**4. Do you smoke tobacco (cigarettes or loose tobacco)?**

*Select only one answer*

- Yes
- No

**5. If yes, how many cigarettes do you smoke per day?**

**6. If yes, how many years have you been smoking?**

**7. Have you ever smoked regularly in the past (even if you quit)? ***

*Select only one answer*

- No
- Yes

**8. If you quit, how many cigarettes did you smoke per day?**

**9. If you quit, how many years did you smoke regularly before stopping?**

**10. Do you smoke heated tobacco products (e.g., IQOS)? ***

*Select only one answer*

- Yes
- No

**11. If yes, how many heated tobacco sticks do you smoke per day?**

**12. If yes, how many years have you been using heated tobacco?**

**13. Do you use electronic cigarettes? ***

*Select only one answer*

- Yes
- No

**14. If yes, what nicotine dosage do you usually use?**

*Select only one answer*

- Nicotine-free
- <5 mg/mL
- 5-10 mg/mL
- 10 mg/mL
- I don’t know
- Other: ________

**15. If yes, how many mL do you vape per day?**

*Select only one answer*

- Less than 2 mL
- 2-4 mL
- 4-8 mL
- More than 8 mL
- I don’t know
- Other: ________

**16. Do you ever smoke both tobacco and electronic cigarettes? ***

*Select only one answer*

- Yes
- No

**17. What are the reasons you started using devices like heated tobacco or electronic cigarettes? *(You can select multiple options)***

*Select all that apply*

- To reduce or quit smoking traditional cigarettes
- Out of curiosity
- Because I think it’s less harmful than traditional tobacco
- Because it’s cheaper
- Because I can use it indoors
- To reduce the impact of second-hand smoke on others
- Other: ________

**18. Have you ever tried to quit smoking? ***

*Select only one answer*

- Yes
- No
- I have never smoked

**19. Have you ever tried accessing a smoking cessation service? ***

*Select only one answer*

- Yes
- No
- I have never smoked

**20. Has your infectious disease specialist ever referred you to a smoking cessation center? ***

*Select only one answer*

- Yes
- No
- I have never smoked

**21. Has your healthcare provider ever prescribed tests related to smoking?**

*Select only one answer*

- Yes
- No
- I have never smoked

**22. If yes, which tests? *(You can select multiple options)***

*Select all that apply*

- Pulmonology consultation
- Spirometry
- Chest X-ray
- Chest CT scan
