# Supplementary material for: Use of Tobacco and Alternative Nicotine Products Among People with HIV: A Cross-Sectional Multicenter Survey
Source: AIDS Behav. 2025 Nov 13;30(4):1088–96. doi: 10.1007/s10461-025-04958-7 (PMC13076392; doi:10.1007/s10461-025-04958-7)
Supplement: Supplementary file 2 — Supplementary Material 2 [file 10461_2025_4958_MOESM2_ESM.docx]

## Table S1. Dunn’s Pairwise Comparison of Pack-Years Across Age Groups

| **Comparison** | **Median (IQR)** | **Z-Value** | **P-Value** |
| --- | --- | --- | --- |
| 20-30 vs 31-40 | 3.5 (2.5-5) vs 7.5 (3.75-12.5) | -2.4250 | 0.109 |
| 20-30 vs 41-50 | 3.5 (2.5-5) vs 12.125 (5-20) | -4.5460 | <0.001 |
| 20-30 vs 51-60 | 3.5 (2.5-5) vs 20 (10-30) | -7.4659 | <0.001 |
| 20-30 vs <20 | 3.5 (2.5-5) vs 2.125 (2-3.75) | 0.1256 | 1 |
| 20-30 vs >60 | 3.5 (2.5-5) vs 25.625 (15-40) | -9.0358 | <0.001 |
| 31-40 vs 41-50 | 7.5 (3.75-12.5) vs 12.125 (5-20) | -3.0402 | 0.018 |
| 31-40 vs 51-60 | 7.5 (3.75-12.5) vs 20 (10-30) | -7.6652 | <0.001 |
| 31-40 vs <20 | 7.5 (3.75-12.5) vs 2.125 (2-3.75) | 1.4565 | 0.677 |
| 31-40 vs >60 | 7.5 (3.75-12.5) vs 25.625 (15-40) | -10.1000 | <0.001 |
| 41-50 vs 51-60 | 12.125 (5-20) vs 20 (10-30) | -5.1007 | <0.001 |
| 41-50 vs <20 | 12.125 (5-20) vs 2.125 (2-3.75) | 2.5339 | 0.081 |
| 41-50 vs >60 | 12.125 (5-20) vs 25.625 (15-40) | -7.9547 | <0.001 |
| 51-60 vs <20 | 20 (10-30) vs 2.125 (2-3.75) | 3.9884 | <0.001 |
| 51-60 vs >60 | 20 (10-30) vs 25.625 (15-40) | -3.2581 | 0.008 |
| <20 vs >60 | 2.125 (2-3.75) vs 25.625 (15-40) | -4.8072 | <0.001 |

## Table S2. Dunn’s Pairwise Comparison of Pack-Years Across Gender Categories

| **Comparison** | **Median (IQR)** | **Z-Value** | **P-Value** |
| --- | --- | --- | --- |
| Cisgender women vs Transgender women | 15.0 (5.7-29) vs 8.75 (5.5-19.25) | 1.4261 | 0.551 |
| Cisgender women vs Non-Binary individuals | 15.0 (5.7-29) vs 14.5 (2.5-29.25) | 0.8284 | 0.897 |
| Cisgender women vs Cisgender men | 15.0 (5.7-29) vs 15.0 (7.0-30.0) | -0.6026 | 0.959 |
| Cisgender women vs Transgender men | 15.0 (5.7-29) vs 0.1 (0.1-0.1) | 1.7097 | 0.360 |
| Transgender women vs Non-Binary individuals | 8.75 (5.5-19.25) vs 14.5 (2.5-29.25) | -0.5107 | 0.974 |
| Transgender women vs Cisgender men | 8.75 (5.5-19.25) vs 15.0 (7.0-30.0) | -1.6646 | 0.388 |
| Transgender women vs Transgender men | 8.75 (5.5-19.25) vs 0.1 (0.1-0.1) | 1.3480 | 0.606 |
| Non-Binary vs Cisgender men individuals | 14.5 (2.5-29.25) vs. 15.0 (7.0-23.0) | -1.0763 | 0.781 |
| Non-Binary vs Transgender men individuals | 14.5 (2.5-29.25) - 0.1 (0.1-0.1) | 1.5048 | 0.496 |
| Cisgender men vs Transgender men | 15.0 (7.0-30.0) vs 0.1 (0.1-0.1) | 1.7590 | 0.330 |

**Table S3. HTPs’ stick used per day according to traditional tobacco habits in people with HIV**

| **Comparison** | **Median** | **Z-Value** | **P-Value** |
| --- | --- | --- | --- |
| Never smoke vs. Current smoker | 7.5 (5-15) vs. 10 (5-15) | -0.19297 | 0.808 |
| Never smoke vs. Former smoker | 7.5 (5-15) vs. 10 (10-20) | -1.155222 | 0.328 |
| Current smoker vs. Former smoker | 10 (5-15) vs. 10 (10-20) | -2.183509 | 0.043 |

## Table S4. HTP Use in PWH divided by Age

| **Age** | **HTP Non-User** | **HTP User** | **Total** |
| --- | --- | --- | --- |
| < 20 years | 7 (50.00%) | 7 (50.00%) | 14 |
| 20-30 years | 36 (72.00%) | 14 (28.00%) | 50 |
| 31-40 years | 106 (76.81%) | 32 (23.19%) | 138 |
| 41-50 years | 202 (88.99%) | 25 (11.01%) | 227 |
| 51-60 years | 325 (93.12%) | 24 (6.88%) | 349 |
| More than 60 years | 290 (95.71%) | 13 (4.29%) | 303 |
| Total | 966 (89.36%) | 115 (10.64%) | 1081 |

## Table S5. E-cigarettes use in PWH divided by Age

| **Age** | **E-cigarettes Non-User** | **E-cigarettes User** | **Total** |
| --- | --- | --- | --- |
| < 20 years | 9 (64.29%) | 5 (35.71%) | 14 |
| 20-30 years | 37 (74.00%) | 13 (26.00%) | 50 |
| 31-40 years | 95 (68.84%) | 43 (31.16%) | 138 |
| 41-50 years | 182 (80.18%) | 45 (19.82%) | 227 |
| 51-60 years | 312 (89.40%) | 37 (10.60%) | 349 |
| More than 60 years | 274 (90.43%) | 29 (9.57%) | 303 |
| Total | 909 (84.09%) | 172 (15.91%) | 1081 |

## Table S6. HTP Use in PWH divided by Gender

| **Gender** | **HTP Non-User** | **HTP User** | **Total** |
| --- | --- | --- | --- |
| Cisgender women | 266 (88.67%) | 34 (11.33%) | 300 |
| Transgender women | 21 (70.00%) | 9 (30.00%) | 30 |
| Non-binary individuals | 18 (66.67%) | 9 (33.33%) | 27 |
| Cisgender men | 659 (91.27%) | 63 (8.73%) | 722 |
| Transgender men | 2 (100.00%) | 0 (0.00%) | 2 |
| Total | 966 (89.36%) | 115 (10.64%) | 1081 |

## Table S7. E-cigarettes use in PWH divided by Gender

| **Gender** | **E-Cigarettes Non-User** | **E-Cigarettes User** | **Total** |
| --- | --- | --- | --- |
| Cisgender women | 258 (86.00%) | 42 (14.00%) | 300 |
| Transgender women | 23 (76.67%) | 7 (23.33%) | 30 |
| Non-binary individuals | 18 (66.67%) | 9 (33.33%) | 27 |
| Cisgender men | 609 (84.35%) | 113 (15.65%) | 722 |
| Transgender men | 1 (50.00%) | 1 (50.00%) | 2 |
| Total | 909 (84.09%) | 172 (15.91%) | 1081 |
